# Characterization of Axon Damage, Neurological Deficits, and Histopathology in Two Experimental Models of Intracerebral Hemorrhage

**DOI:** 10.3389/fnins.2018.00928

**Published:** 2018-12-11

**Authors:** Yao Liu, Gang Lu, Xian Wei Su, Tao Ding, Wen Li Wang, Yong Mei Li, Wai Sang Poon, Li Juan Ao

**Affiliations:** ^1^School of Rehabilitation, Kunming Medical University, Kunming, China; ^2^Division of Neurosurgery, Department of Surgery, Prince of Wales Hospital, The Chinese University of Hong Kong, Shatin, Hong Kong; ^3^School of Biomedical Sciences, The Chinese University of Hong Kong, Shatin, Hong Kong; ^4^Rehabilitation Department, The First Affiliated Hospital of Kunming Medical University, Kunming, China; ^5^Rehabilitation Department, The Second Affiliated Hospital of Kunming Medical University, Kunming, China

**Keywords:** internal capsule, intracerebral hemorrhage, experimental, white matter, damage

## Abstract

Spontaneous intracerebral hemorrhage (ICH) is one of the most lethal forms of stroke. From the limited previous studies and our preliminary data, white matter is considered a key predictor of the outcome and potential target of recovery. The traditional ICH model induced by injection of autologous blood or bacterial collagenase into striatum (ST) demonstrated a spontaneous functional recovery within one or 2 months. We hypothesis that an internal capsule (IC) lesion might lead to long-term axonal damage and long lasting functional deficits. Thus in this study, a modified internal capsule ICH model was conducted in rats, and the axonal damage, neurological deficits, histopathology as well as electrophysiology were characterized. The finding demonstrated that compared to ST group, the modified IC lesioned model exhibited a relatively smaller lesion volume with consistent axonal loss/degeneration and long-lasting neurological dysfunction at 2 months after ICH. Functionally, the impairment of the mNSS, ratio of contralateral forelimb usage, four limb stand index, contralateral duty cycle and ipsilateral SSEPs amplitude remained significant at 56 days. Structurally, the significant loss of PKCγ in ipsilateral cortical spinal tracts of IC group and the consistent axonal degeneration with several axonal retraction bulbs and enlarged tubular space was observed at 56 days after ICH. This study suggested that a modified IC lesioned model was proved to have long lasting neurological deficits. A comprehensive understanding of the dynamic progression after experimental ICH should aid further successful clinic translation in animal ICH studies, and provide new insights into the role of whiter matter injury in the mechanism and therapeutic targets of ICH.

## Introduction

Spontaneous intracerebral hemorrhage (ICH) is the most lethal subtypes of stroke with a two times higher rate of incidence in Asian people, results in 30–50% mortality (Feigin et al., 2009) and only 10–20% of all survivors remaining independent after 6 months from onset (van Asch et al., 2010). Considerable preclinical studies have been conducted to investigate the pathophysiology and neuroprotective interventions of ICH. However, to date, none of the preclinical research that proven positive results has successfully translated in clinical setting. This failure has occurred for several reasons that mainly in poor modeling, lack of behavioral testing and inadequate experimental design (Manaenko et al., 2011).

The rodent ICH model induced by the injection of autologous blood or bacterial collagenase into the basal ganglia striatum (ST) has been widely used since the 1980s (Rosenberg et al., 1990) and served as the classical and reproducible ICH animal model in most preclinical studies (Nath et al., 1986; MacLellan et al., 2010; Rosenberg, 2011). In recent years, based on the pathophysiology of ICH and injury progression, modified ICH animal models have been developed to more closely mimic the clinical events of ICH in human patients. Although the value of the different modified models remains controversial, the numerous studies and reviews have reached a consensus that an appropriate and reproducible model should be selected based on a specific study purpose and to achieve a more reliable clinical translation.

The etiology of human spontaneous ICH is a rupture of the small vessel arterial walls, which are probably weakened by the effects of chronic hypertension, amyloid angiopathy, vascular malformations, and additional vascular risk factors. The most common sites are the deep cerebral regions, basal ganglia and thalamus (Flaherty et al., 2006). The most widely affected functional structures are the white fibers of the motor system, which are known as the internal capsule (IC) and run alongside the basal ganglia. Functional disabilities after hemorrhagic stroke result not only from neuron or synapse losses, but also from primary damage to the white matter axons. Early animal studies indicated that axon degeneration can be triggered by even brief focal ischemia (Pantoni et al., 1996), neurotoxins (Cliffer et al., 1998), inflammatory mediators (Neumann, 2003), and defects in transport, myelin and oxygen delivery (Coleman and Perry, 2002). Based on the evidence of previous animal studies, blood–brain barrier (BBB) damage and the serum proteins or plasma component following ICH lead to white matter injury, even before neuronal necrosis and apoptosis (Wagner et al., 2002).

In contrast to human beings, the rodent ST is a large structure capable of containing hematomas (e.g., 50 to 100 μl) relative in size to large hematomas in a human patient. However, rats have a low mortality rate and a mild sensorimotor deficit (MacLellan et al., 2012). In addition, the relative paucity of white matter in the rodent ST weakens the lesion effect on the animal’s motor functions. However, white matter damage is considered a key predictor of the outcomes of human neurological disorders including stroke, brain trauma, and spinal cord injury. Two published studies recently investigated small hematomas and hemorrhagic lesions near the IC and reported relatively severe motor dysfunction (Masuda et al., 2007a,Masuda et al., 2007b). The study of white matter damage in stroke models is worthwhile; particularly the axonal degeneration and regeneration present in the ICH models.

As such, this study established a modified IC-ICH model for rats, and characterized the relevant axon damage, neurological deficits, and histopathology.

## Materials and Methods

### Animal Preparation and Intracerebral Infusion

Our animal use protocols were approved by the Guide for the Animal Care and Use Committee of the Chinese University of Hong Kong. Ninety adult male Sprague-Dawley rats (250–300 g) were used. All of the animals were housed under a 12-h light/12-h dark cycle and given free access to food and water. The intracerebral infusion was performed as described in previous report (Liu et al., 2013). In brief, the rats were anesthetized via the intraperitoneal administration of ketamine (100 mg/kg) and xylazine (10 mg/kg), placed in the prone position and secured on a stereotaxic frame. The head hair was shaved before an incision was made on the scalp. A 26-gauge Hamilton syringe needle was inserted through a 1-mm burr hole into the right ST of each rat in the ST group at 3.0 mm lateral and 0.2 mm anterior to the bregma and 6.0 mm deep. According to Masuda’s methods with some modifications (Masuda et al., 2007a), the needle was injected into the right ST and near the IC of the rats in the IC group at 3.7 mm lateral and 2.0 mm posterior to the bregma and 6.0 mm deep. Type IV bacterial collagenase (1.2 μL, Sigma, C5138 0.2 5U in 1 μL 0.9% NaCl) was infused into the ST and IC groups using a microinfusion pump. After injection, the syringe was left in position for 10 min to prevent back-leakage before being withdrawn. The borehole was then sealed with bone wax, the incision was sutured and the animals were kept warm and allowed to recover from the anesthesia. Sham group received the same volume of saline infusion in the right striatum. Their well-being was assessed according to their body weight, ambulation normality, feeding, and grooming.

### Neurological Outcome Measurements

**FIGURE 1:**
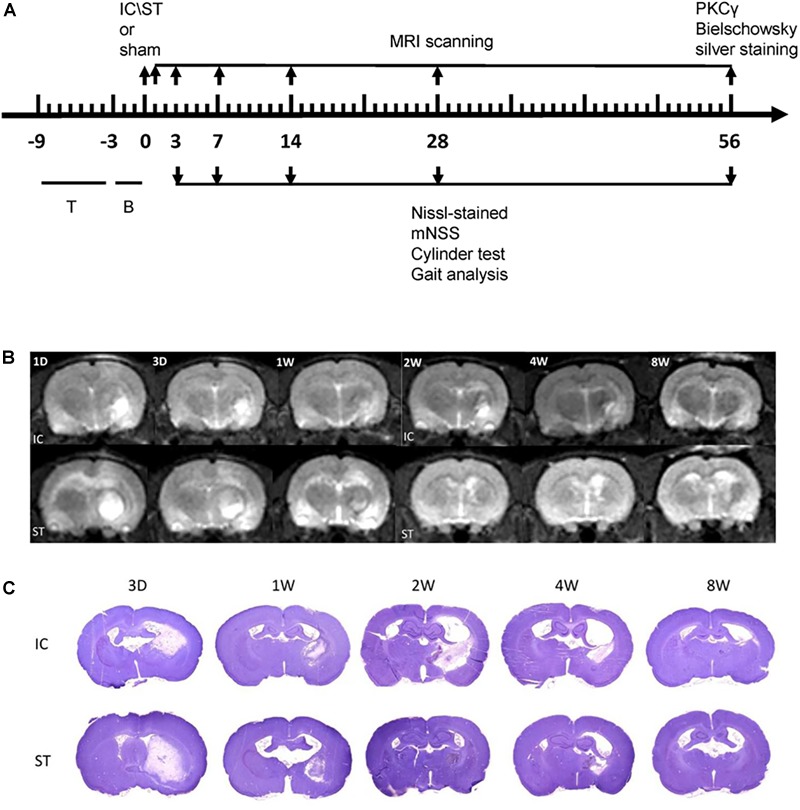
**(A)** Schema illustrating the time course of testing procedures. Numbers indicate days relative to the two modeling ICH or sham surgery. T, training; B, baseline evaluations. **(B)** A representative image from MRI scanning at the level of maximum hematoma diameter from day 1 to day 56 following two ICH modeling. T2W1 of corresponding regions are shown. **(C)** A representative Nissl-stained image on day 3, 7, 14, 28, and 56 of the two groups with a cross section through the entire brain.

Throughout the experiment, the animals were not subjected to any undue stress or irritation, and their condition and wellbeing were monitored. All of the behavioral training and testing were carried out in a quiet room at a fixed time during the animal’s light phase by at least two experimenters who were blinded to experimental group. Baseline evaluations were conducted 2 days prior to surgery. Figure 1A illustrates the time course of all neurological outcome measurements, with the time points of the testing procedures described in the figure legend.

### Functional Assessment

The modified neurologic severity score (mNSS) is a composite of motor, sensory (visual, tactile, and proprioceptive), balance and reflex tests. Neurologic function is graded on a scale of 0 to 18, with 0 indicating normal neurologic function and 18 indicating maximum functional deficit. In assessing injury severity, one point is awarded when the animal is unable to perform a test or lacks a tested reflex (the higher the score and the more severe the injury) (Otero et al., 2011). The rats were placed individually into a transparent cylinder 20 cm in diameter and 45 cm high, and videotaped from the side for 5 min using a mirror. The forelimb use asymmetry test (Cylinder test) (Schallert et al., 2000) was used to assess their sensorimotor function and test the instances of spontaneous forelimb use. It calculated the initial placement of a forelimb on the wall and subsequent movements along the wall. This calculation was detailed in a previous report (Liu et al., 2013).

For the locomotion assessment, gait analysis was conducted using the CatWalk system (Noldus Information Technology, Wageningen, Netherlands) (Koopmans et al., 2005; Liu et al., 2013). After 1 week of training prior to injury, the rats were put in a meter-long glass plate with a fluorescent light beamed into the glass walkway floor from one side and a high-speed video recorder mounted under the glass. As the rats ran along the walkway without any interruption or hitch in a dimly lit environment, the light was reflected downward and captured by the video recorder. A large number of locomotion parameters were then analyzed automatically using the CatWalk XT software (version 10). A minimum of three correct crossings per animal was required for each time point (Hamers et al., 2001; Koopmans et al., 2005).

### Electrophysiological Recording

#### Somatosensory Evoked Potential (SSEP)

In-clinic somatosensory evoked potentials (SSEPs) comprise an objective approach to evaluating the integrity of the CNS sensory pathways (Pereon et al., 1995; Feys et al., 2000). In animal studies, SSEP techniques have been applied to evaluate the functional outcome of experimental-stroke-induced white matter injury (Lee et al., 2004; Chen et al., 2011). Each rat was anesthetized and secured carefully onto the stereotaxic frame in a horizontal position. The head hair was shaved and the skin was disinfected and rinsed with 70% ethanol. A 1-cm-long midline incision was made over the scalp and the cranium was exposed. The stereotactic coordinates were used to locate the right sensorimotor cortex at 1.5 mm posterior and 2.5 mm lateral to the bregma. A 1-mm borehole was then carefully drilled into the skull under the surgical microscope without destroying the dura mater and cortex. A metal microelectrode was positioned above the dura and connected to the Nicolet Endeavor CR IOM System. A needle electrode was placed under the skin of the nose and served as a reference. Another needle electrode was placed in the tail as a ground reference. A pair of stimulators was placed under the skin of the left hind limbs contralateral to the recording side. The somatosensory stimuli consisted of a 3-mA current with 2.1-Hz repetition rates and a 300-μs pulse width. The SSEP signals were filtered with band-pass (10–2,000 Hz) and notch (60 Hz) filters and recorded 50 evoked potentials on average. The average SSEP records were stored and analyzed digitally by a computer using the Nicolet Endeavor CR IOM System software. All of the signals were recognized, as the waveforms consisted of the first positive (P1) and negative (N1) peaks. The N1 latency and P1 to N1 amplitude were measured.

### Measurement of Tissue Loss Volume

The hemorrhagic tissue loss volume was measured as previously described using both magnetic resonance imaging (MRI) and histological staining (Nissl staining) imaging from 3 days to 56 days after onset. MRI was performed using a 3-T clinical scanner (Achieva, Philips Healthcare, Best, Netherlands) under anesthesia. A custom-made quadrature RF volume coil (internal diameter = 7 cm) was used as the signal transmitter and receiver, and 13 contiguous coronal T2-weighted images were used to cover the entire rat brain. Four slices were used to evaluate the haematoma and volume of injury (slice thickness = 2.0 mm, slice gap = 0.2 mm.), and the images were analyzed with a Philips DICOM Viewer.

For the histological sections, the rats were transcardially perfused with 0.9% saline followed by 4% paraformaldehyde (PFA) in phosphate buffered saline (PBS). Their brains were then fixed in 4% PFA and dehydrated in 15%, 20% and finally 30% sucrose. 40-μm cryostat-processed tissue sections were taken every 200 μm through the entire brain and stained with cresyl violet. Histological analysis was performed using the Spot Imaging System.

Two researchers who were blinded to the treatment condition calculated the tissue loss volume via histological and MRI analysis using the following equations (MacLellan et al., 2006):

Volume of tissue loss = remaining volume of normal hemisphere – remaining volume of injured hemisphere, and

Volume of hemisphere = average (area of complete coronal section of the hemisphere – area of ventricle – area of damage) × interval between sections × number of sections.

### Measurement of Axonal Degeneration/Loss

#### Silver Staining

Bielschowsky silver staining was performed to visualize the axonal pathology (Chen et al., 2010; Cui et al., 2010). In brief, after transcardial perfusion and post-fixation in 4% PFA, the brains were embedded in paraffin. A series of 8-μm-thick coronal and sagittal sections were cut from the paraffin block. The slices were immersed in 20% silver nitrate for 2 h in the dark and then washed in reducer for 5 min. They were then placed in an ammoniacal silver solution for 1–2 min. After being washed in a constant flow of reducer and distilled water, the slices were fixed in 5% sodium thiosulphate, dehydrated, and mounted.

#### Protein Kinase C Gamma Type (PKCγ) Immunostaining

PKCγ has been widely used as a marker to assess the structural status of corticospinal tracts (CST) in experimental animal models. The animals were perfused with 0.9% saline followed by 4% PFA in 0.1M PB. The brain and brain stem tissues were post-fixed in 4% PFA at least overnight, and then dehydrated and paraffin embedded. A series of 5-μm-thick coronal and sagittal sections were cut from the paraffin block. The sections were dewaxed in xylene and washed in gradient alcohol and distilled water, and then heated in a 10-mM sodium citrate buffer (pH 6.0) for 10 min and cooled for 10 min. The sections were then rinsed with triton phosphate buffered saline (PBST, pH 7.2–7.4) three times at 5 min each, and immersed in 3% H_2_O_2_ methanol for 10 min to block the endogenous peroxidase. The sections were rinsed with PBST three times and blocked with 10% normal blocking serum with triton for 1 h at room temperature. They were then directly incubated with the primary antibody diluted with 5% normal serum (rabbit anti-PKCγ antibody, Abcam, #ab71558) at a concentration of 1:100 overnight at 4°C. After being washed with PBST three times at 10 min each, the sections were incubated for 2 h at room temperature with the secondary antibody diluted with 5% normal serum (biotinylated anti-rabbit IgG, Vector Lab, #BA-1000) at a concentration of 1:200. They were then placed in DAB for 5–10 min to visualize the peroxidase complex. After color development, the sections were mounted in DPX (Sigma-Aldrich #06522, MO, United States). A negative control was performed using normal serum instead of the primary antibody, and no positive staining was observed under this condition.

#### Western Blotting

Right medullary corticospinal tract tissues were collected from the two groups at the same time points. Cortex tissue samples (weighing about 50 mg) were homogenized using an adapter pellet pestle motor (Sigma-Aldrich #Z359971, MO, United States) in 200 μl of ice-cold lysis buffer. The homogenate was centrifuged at 14,000 rpm for 30 min at 4°C, and the supernatant was transferred into a clean tube and stored at -80°C. The protein concentration of the extract was determined using a DC protein assay kit (Bio-Rad, Hercules, CA, United States). The sample concentration was calculated and adjusted to 10 μg/μl using distilled water. Equal amounts of the sample protein (40 μg) from different time points were boiled in the mixture with a sixfold loading buffer for 10 min at 100°C and then loaded into the wells of 10% Sodium Dodecyl Sulfate PolyAcrylamide Gel Electrophoresis (SDS-PAGE). Electrophoresis was performed at 60 V for 30 min in stacking gel and 100 V for 60 min in separating gel, followed by simultaneous electrophoretic transfer (Mini Trans-Blot Cell, Bio-Rad) to the nitrocellulose membrane (Bio-Rad) at 85 V for 100 min. The membrane was then blocked for 1 h at room temperature in the Triton X-100 (TBST) containing 5% non-fat milk and then incubated for at least 2 h at room temperature or overnight at 4°C with the primary antibody for PKCγ (1:1000; Abcam, #ab71558) at the appropriate dilution in TBST containing 5% non-fat milk. After washing with TBST three times at 10 min each, the membrane was incubated for 2 h at room temperature. The secondary antibody (Anti-rabbit IgG; 1:2000; Santa Cruz, #sc-2004) was diluted in TBST containing 5% non-fat milk and then washed in TBST three times. The target protein bands were visualized by adding enhanced chemiluminescent reagents (ECL Kit, Thermo Fisher Scientific, #32106) and exposing them to ECL hyperbond film (Fuji Medical X-Ray Film, #M001, Japan). The density of the specific immunoreactive bands was determined using digital images and the Quantity One software (Bio-Rad). The housekeeping gene Glyceraldehyde 3-phosphate dehydrogenas (GAPDH) was used as an internal control.

### Statistical Analysis

All of the data presented were calculated as the mean ± standard error of the mean. All of the behavioral tests were carried out by at least two experimenters who were blinded to the group identity. The behavioral scores for the Cylinder test and mNSS were analyzed using repeated-measures analysis of variance (ANOVA) (SPSS 17.0; SPSS Inc., Chicago, IL, United States), followed by one-way ANOVAs or the Mann–Whitney *U* test to analyze the differences between the groups at a given time point. The optical density of Western blot band was analyzed using one-way ANOVAs followed by *post hoc* Bonferroni tests were used to analyze the difference. A *p*-value of 0.05 was considered the threshold for significant difference.

## Results

### Extent of Cerebral Hemorrhage After IC Lesioned ICH

The haematoma in the IC group (*n* = 6 at each time point) extended from +1.0 to -3.0 mm anterior to the bregma and approached the IC range (Figure 1B). The volume of tissue loss in the IC group, measured using both MRI and histology sections (Figure 1C), was smaller than that of the ST group (*n* = 6 at each time point) on days 3, 7, and 56 (Table 1). The variation in tissue loss volume measured in the ST group was well described in our previous report (Liu et al., 2013). The volume of tissue loss in the IC group was greatest on day 3, decreased on day 7, and increased in the second peak value on day 14 after onset, then decreased over time. In the first month after ICH, tissue loss volume decreased in line with hematoma resolution (Matsushita et al., 2013). However, in IC lesioned model, the tissue loss volume increased in line with not only edema and the mass effect of the hematoma, but also white matter degeneration, according to those results described in Masuda et al. (2007a).

**Table 1:** Tissue loss volume determined by MRI scanning and Nissl staining images in two ICH models.

	MRI				
	3D	7D	14D	28D	56D
IC	36.85 ± 4.37	13.21 ± 0.95	36.45 ± 4.13	32.01 ± 3.15	19.09 ± 2.54
ST	73.82 ± 1.45	41.20 ± 2.36	39.17 ± 1.17	25.71 ± 2.05	36.11 ± 2.48
	**Histology**				
IC	56.10 ± 3.60	30.94 ± 3.19	63.85 ± 4.13	40.05 ± 3.87	14.91 ± 1.11
ST	73.18 ± 1.77	35.59 ± 2.30	20.81 ± 1.33	22.63 ± 1.14	25.71 ± 1.54

### Neurological Functions After IC Lesioned ICH

**FIGURE 2:**
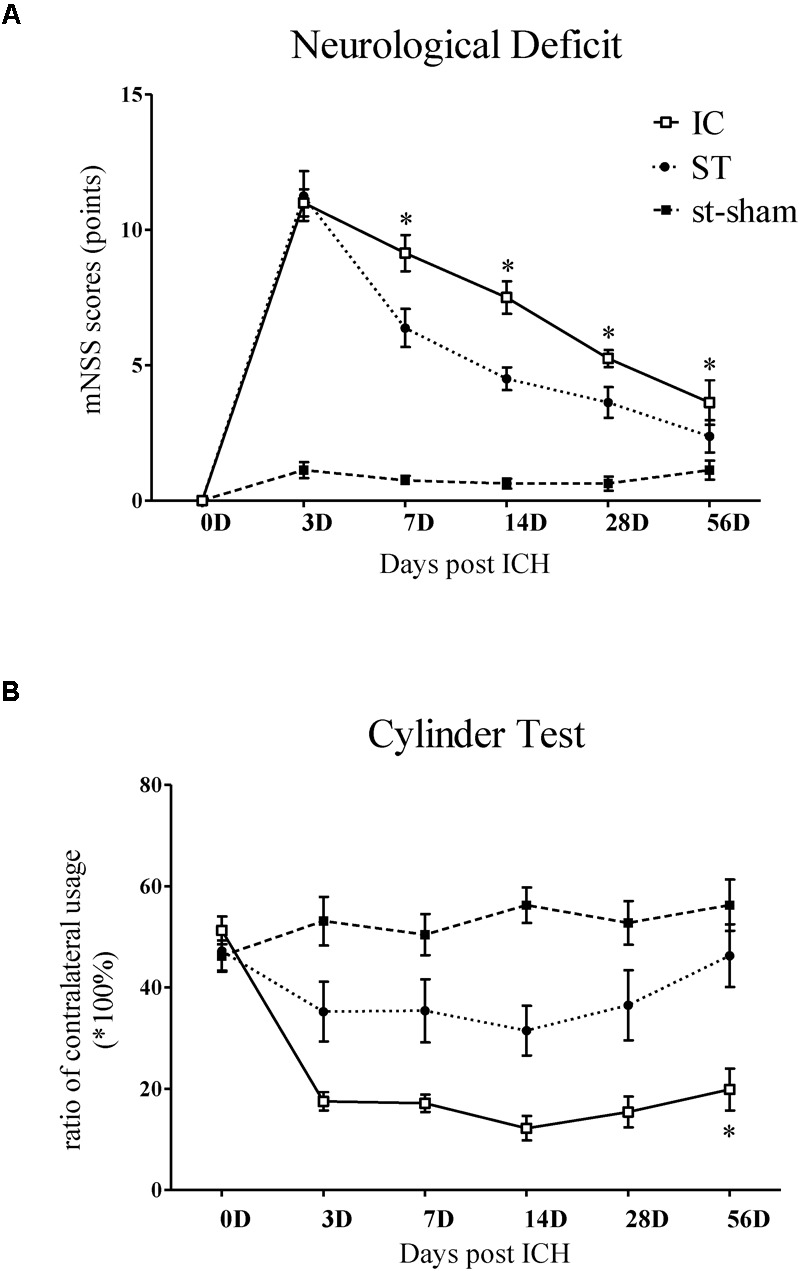
Representative mNSS score and Cylinder test of IC model over 2 months. The mNSS score **(A)** was shown as the points and Cylinder test (forelimb asymmetry use) was shown as the percentage of wall contacts made with the contralateral forelimb **(B)** in two ICH models and sham groups (*n* = 10 in each group, data are shown as mean ± SEM ^∗∗∗^*p* < 0.001, ^∗∗^*p* < 0.01, ^∗^*p* < 0.05; ^∗^IC group was compared with ST group at the same time point value.

The mNSS of the IC group (*n* = 10 in each group) increased and peaked on day 3 and declined from days 7 to 56 (Figure 2A) compared with the baseline. A similar trend was also observed in the ST group. Compared with the ST group, the animals in the IC group showed significant increasing scores, indicating prolonged neurologic dysfunction at the same time points from days 7 to 56 (Figure 2A, ^∗^*p* < 0.05). No difference was observed between the two groups on day 3.

The Cylinder test affected contralateral forelimb use during wall exploration, revealing a reduction in the IC group (*n* = 10 in each group) from days 3 to 56 (Figure 2B), with statistical significance on days 28 and 56 compared with the baseline (*p* < 0.001 and *p* < 0.01). A relatively slight reduction in the ST group was observed throughout the whole duration. A significant difference in the two groups was found at a delayed time on day 56 (*p* < 0.05).

#### Gait Analysis

**FIGURE 3:**
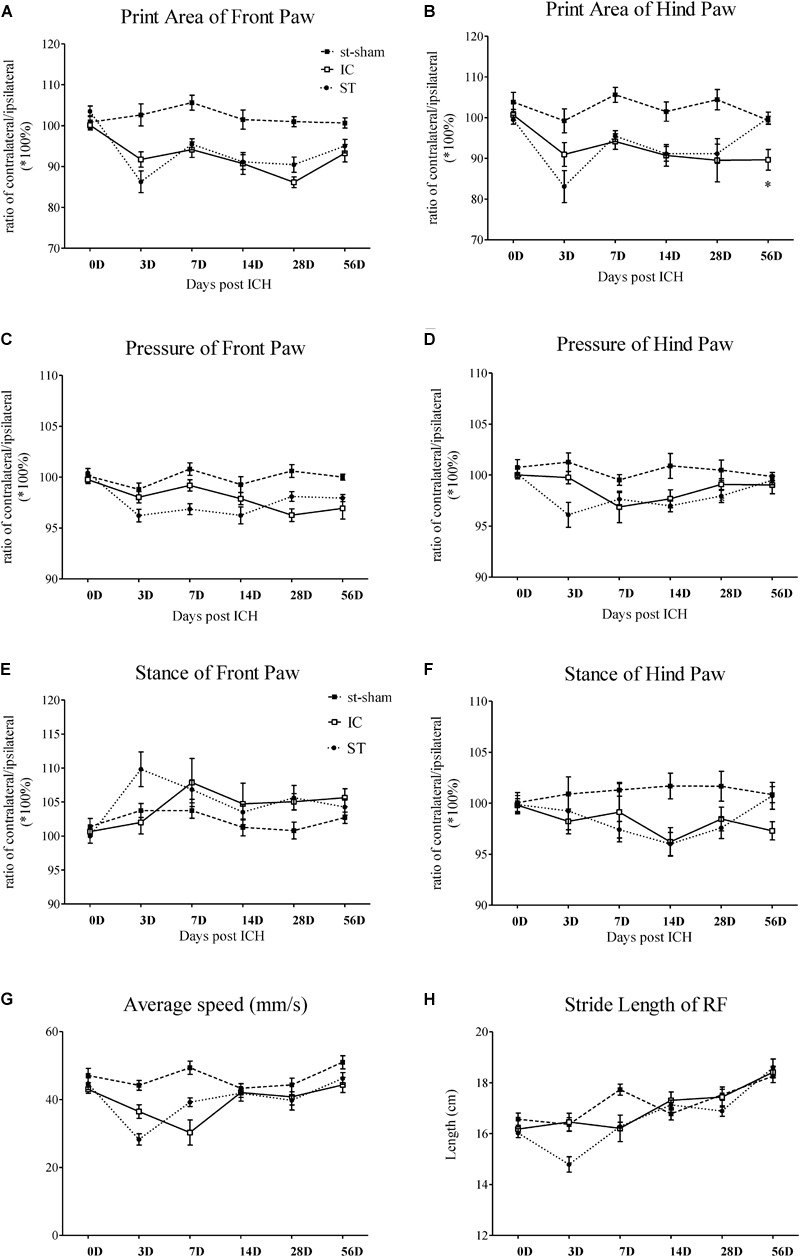
Gait parameter measurements of IC model over 2 months. Print area and paw pressure in contralateral LF **(A,C)**, LH **(B,D)** decreased in two ICH groups (*n* = 10 in each group). The stance phase of LF in IC group increased when compared to that of the ipsilateral forepaw (right forepaws, RF) on day 7 and day 28 **(E)**. The stance phase of LH decreased on day 14 **(F)**. The two ICH groups exhibited no significant difference. The time course changes in the average speed **(G)** and stride length in right front limbs **(H)** in two ICH models. Data are shown as mean ± SEM ^∗∗∗^*p* < 0.001, ^∗∗^*p* < 0.01, ^∗^*p* < 0.05, ^∗^IC group was compared with ST group at the same time point value.

The gait parameters of the IC and ST groups (*n* = 10 in each group) were compared from days 3 to 56. Changes to static, dynamic and coordination gait parameters in the IC group are shown in Figure 3. The most significant parameters analyzed using CatWalk are described in further detail in the following paragraphs.

##### Static gait parameters

The individual paw print area revealed an unequal distribution of the two front paws and two hind paws in the two groups (Figures 3A,B). In the IC group, the significant asymmetry pattern of the front paw print area was observed on day 3, gradually worsened and reached the peak value on day 28 (Figure 3A). The contralateral hind paw print area decreased significantly in the two ICH groups. The decrease in the injured hind paw print area of the IC group worsened on days 14, 28, and 56 (Figure 3B). In contrast with the ST group, a prolonged deterioration of the hind paw print area asymmetry was observed in the IC group (Figure 3B, *p* < 0.05).

A contralateral decrease in the pressure of the injured forepaws and hind paws was observed in the two ICH groups (Figures 3C,D). An asymmetry in the forepaw pressure was observed on day 28 in the IC group (Figure 3C), and the deficits lasted until day 56 after onset. The injured hind paw pressure decreased on days 7 and 14 (Figure 3D). Compared with the ST group, no significant prolonged deterioration of the forepaw and hind paw pressure asymmetry was observed in the IC group (Figures 3C,D).

##### Dynamic Parameters

A diagonal increase in stance duration in the LF and right hind paws (RH) was observed in the ICH groups (Figures 3E,F). The ratio between the contralateral (left) and ipsilateral (right) paws exhibited an opposite trend in the front and hind paws. The stance phase of the contralateral LF in response to the IC group injury increased when compared to that of the ipsilateral forepaw (right forepaws, RF) on day 7 and day 28 (Figure 3E). The value of the contralateral LH decreased on day 14 (Figure 3F). The two ICH groups exhibited no significant difference in the hind paw.

#### Average Speed

The average speed of the ST group decreased on day 3 and the speed of the IC group decreased on day 7 after onset (Figure 3G). The two ICH groups exhibited no significant difference in average speed from days 14 to 56 after ICH.

#### Coordination Parameters

A significant decrease was detected in stride length in the ST group. However, the change in stride length (referred to as step length in human beings) in the IC group was not observed during the 2 months after ICH (Figure 3H). No significant difference was observed in the other coordination parameters of the IC group such as the base of support (BOS) and support on style, which changed significantly in the ST group on day 3.

#### Stand Index and Duty Cycle

**FIGURE 4:**
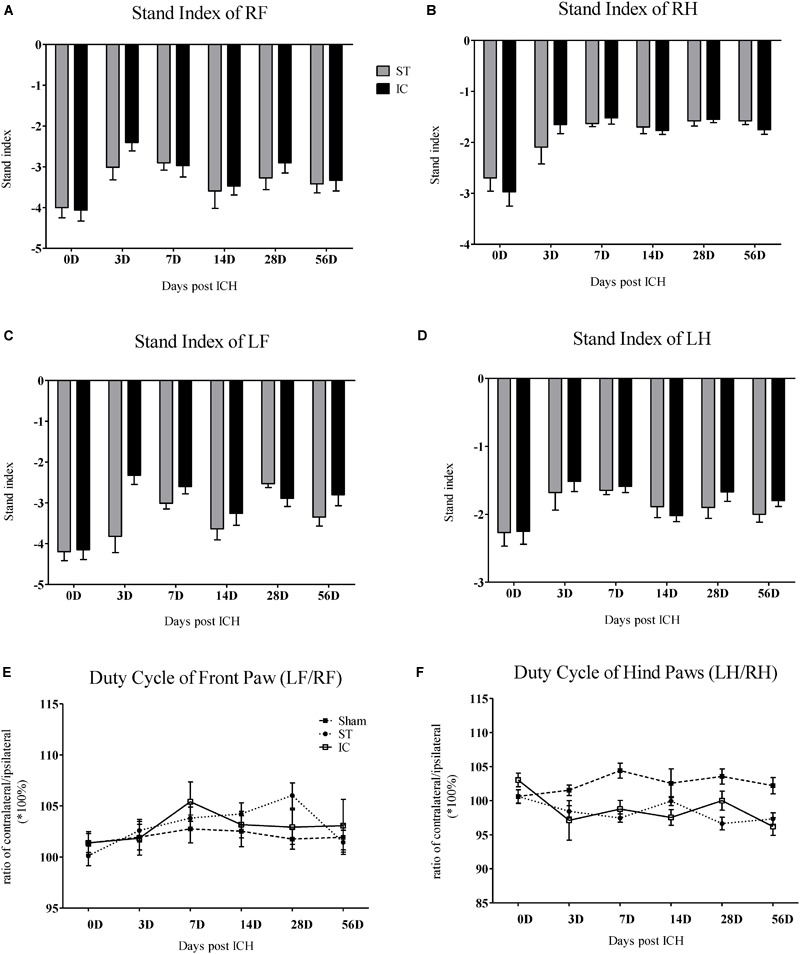
Stand index and duty cycle of IC model over 2 months. The time course changes in the stand index in four limbs **(A–D)** of two ICH models (*n* = 10 in each group). The time course changes in duty cycle in front limbs **(E)** and hind limbs **(F)** of two ICH models (*n* = 10 in each group). Data are shown as mean ± SEM.

The stand index indicates the speed the paw is lifted from the floor (arbitrary units). Impairment in stand index was observed in all four limbs in IC group from day 3 to day 56 (Figure 4). A decreased in the absolute value of stand index of right front paws was observed on days 3, 7, and 28 in animals subject to IC group (Figure 4A). A decrease in stand index of right hind paws was found on days 3, 7, 14, and 56 (Figure 4B). The two ICH groups exhibited no significant difference in stand index from days 3 to 56 after ICH.

The parameter duty cycle measures the percentage of time the paw accounts for the total step cycle of that paw. Animal subjected to ICH displayed a significant decrease in duty cycle in the LH (Figure 4E). The two ICH groups exhibited no significant difference in duty cycle from days 3 to 56 after ICH.

#### Depressant SSEPs

**FIGURE 5:**
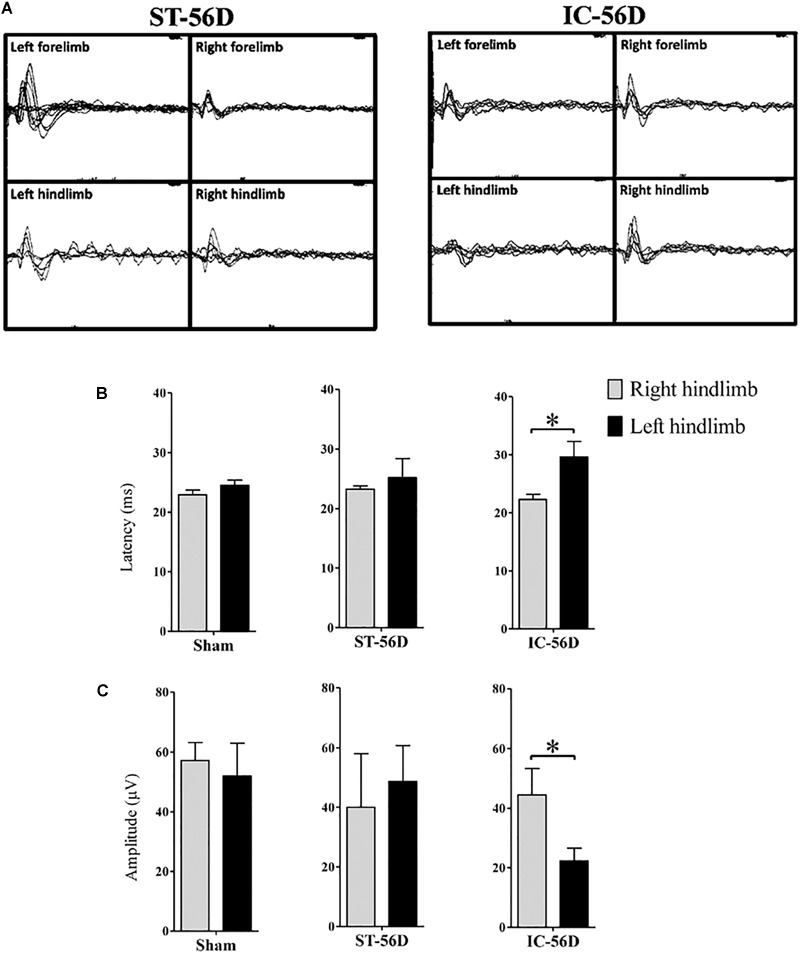
Somatosensory evoked potentials (SSEPs) records of IC model at 2 months. **(A)** Representative examples SSEPs recorded from the right primary cortex and evoked by left fore- or hind limb stimulation in two ICH models at 2 months. Depressant SSEPs of left fore- and hind limb in IC group at day 56 after ICH were identified compared to ST group. **(B)** IC group showed significant delayed SSEP latency in left hind limbs on day 56. **(C)** IC group showed significant depressant SSEP amplitude in left hind limbs on day 56. *N* = 6 in each group. Data are shown as mean ± SEM ^∗∗∗^*p* < 0.001, ^∗∗^*p* < 0.01, ^∗^*p* < 0.05, compared between the two groups at the same time point value.

Depressant SSEPs of left hind limb in IC group (*n* = 6 in each group) were found at day 56 after ICH (Figure 5A). The N1 latency in left hind limbs of IC group was significant delayed compared to right hind limbs but not found in ST group at day 56 (Figure 5B, *p* < 0.05). Statistic comparison of N1 to P1 amplitude between left and right hind paws in two groups showed a significant decrease in hind limbs of IC group than ST group at day 56 (Figure 5C, *p* < 0.05).

### Long-Term Functional Deficits After IC Lesioned ICH

**FIGURE 6:**
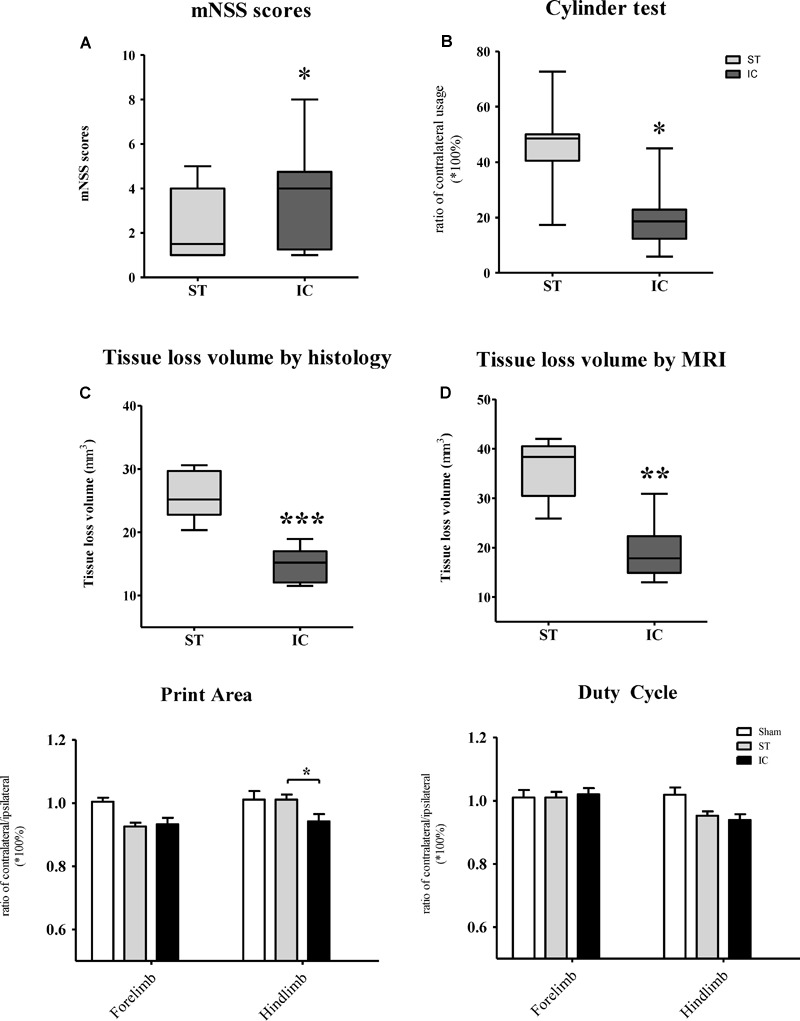
Long-term neurologic deficits, gait deficits, and tissue loss volume in IC model on day 56. The comparison of mNSS scores **(A)** and Cylinder test **(B)** between two ICH models at 56 days post-ICH (*n* = 10 in each group). The comparison of tissue loss volume measured by histological sections **(C)** and MRI scanning **(D)** between two ICH models at 56 days post-ICH (*n* = 6 in each group). Compared to ST group (*n* = 10 in each group), IC group showed significant long lasting gait deficits in print area in fore- and hind limb **(E)**, no significant difference in duty cycle **(F)**. Data are shown as mean ± SEM ^∗∗∗^*p* < 0.001, ^∗∗^*p* < 0.01, ^∗^*p* < 0.05, ^∗^IC group was compared with ST group.

The mNSS scale points of the ST group reached the normal level set by the sham group at 56 days after ICH, with no significant difference. However, the mNSS significantly increased in the IC group at 56 days compared with ST group (Figure 6A, *p* < 0.05). To further explore the forelimb deficits, a Cylinder test was conducted for the two ICH groups at 56 days after ICH. Both group used their contralateral forelimbs less often than the baseline. A significant difference in the ratio of contralateral forelimb usage was observed in the IC group at 56 days after ICH compared with the ST group (Figure 6B, *p* < 0.05).

The gait parameters changed along with the long-term effects in both ICH groups, including in terms of the paw print area and duty cycle. The paw print area of the contralateral forepaws and hind paws decreased in both ICH groups (Figure 6E). The hind paw print area asymmetry in the ST group recovered to the sham group level at 56 days, and that of the IC group differed significantly from that of the ST group (Figure 6E, *p* < 0.05). The gait parameter duty cycle measures the percentage of time the paw accounts for the total step cycle of that paw. Animal subjected to both IC and ST lesion displayed a significant decrease in duty cycle in the left hind paw, however, with no significant different between two groups (Figure 6F).

### Long-Term Haematoma Extent, Neuronal and Axonal Damage After IC Lesioned ICH

The tissue loss volumes of the IC and ST groups (*n* = 6 in each group) were compared on day 56 after ICH. The IC group showed a relatively smaller lesion volume compared with the ST group on day 56 when measured using histological staining sections (Figure 6C, *p* < 0.01) and MRI (Figure 6D, *p* < 0.001).

**FIGURE 7:**
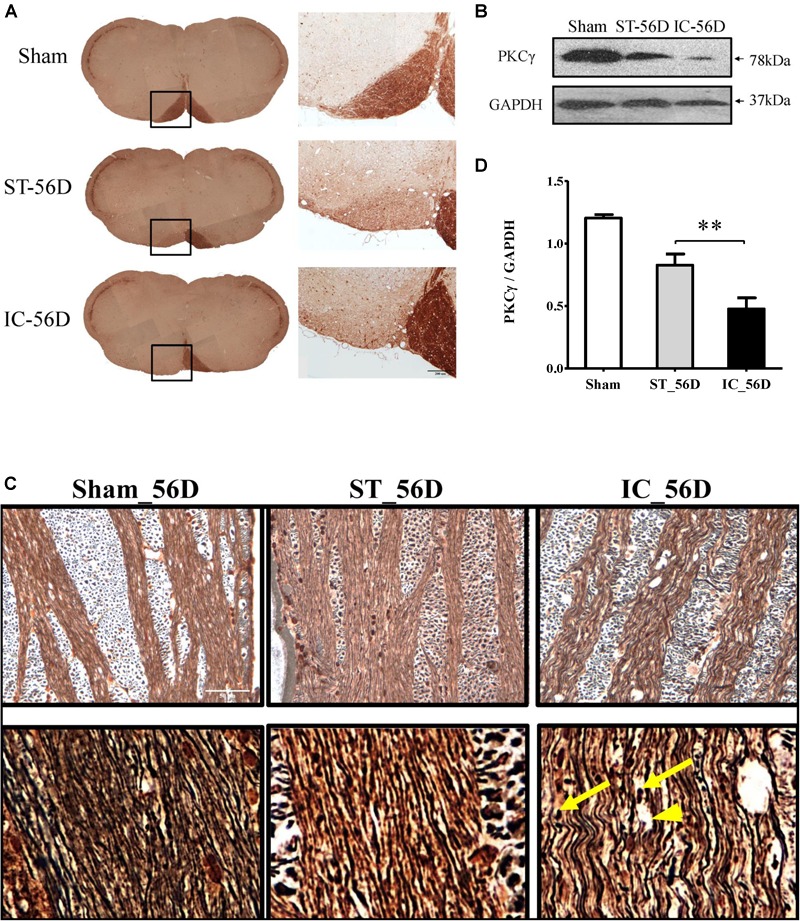
**(A)** Representative immunohistochemistry of PKCγ in medullary CST of IC group and ST group demonstrating significant axonal loss in the ipsilateral pyramid white matter tracts on day 56. **(B)** Quantification of PKCγ levels in CST of three groups on day 56 from western blot, represented as the optical density. **(C)** A significant reduction of PKCγ expression levels in rats with IC at 56 days compared to ST groups (*n* = 6 in each group). Data are shown as mean ± SEM. ^∗∗∗^*p* < 0.001, ^∗∗^*p* < 0.01, ^∗^*p* < 0.05. ^∗^IC group was compared with ST group. Scale bar = 200 μm. **(D)** Comparison of Bielschowsky sliver staining of the descending CST in ST and IC rats at 56 days after onset. Photomicrographs from the longitudinal sections showed axonal retraction bulbs (yellow arrows) with dilated myelin sheaths and enlarged tubular space (arrowhead) in IC group rather than ST group on day 56 after ICH. Scale bar = 50 μm.

On day 56 after ICH, the immunohistochemistry of PKCγ clearly demonstrated a nearly complete loss of PKCγ expression in the ipsilateral medullary CST in IC group (*n* = 6 in each group) (Figures 7A,B). Compared to ST group, the expression levels of PKCγ in IC group significantly decreased on day 56 in the ipsilateral CST (Figure 7C, *p* < 0.01).

The Bielschowsky silver staining positive axons were observed in both IC and ST groups, with a decreased density (Figure 7D). The longitudinal sections of IC group showed axon twisted disrupted with several axonal retraction bulbs (Figure 7D, yellow arrows) and dilated myelin sheaths with enlarged axonal space (Figure 7D, arrowhead) rather than that of ST group on day 56 after ICH, indicating a more severe long-term axonal degeneration in IC group.

### Summary

The IC lesioned ICH model exhibited a relatively smaller lesion volume with consistent axonal loss/degeneration and long-lasting neurological dysfunction at 2 months after ICH. The impairment of the mNSS, ratio of contralateral forelimb usage, asymmetries in the hind paw print area, ipsilateral SSEPs amplitude, and the loss of PKCγ in ipsilateral CST of the IC group exhibited significant differences from the ST group at 56 days.

## Discussion

The clinical translation of prospective neuroprotective agents is probably only successful in therapies that benefit functional improvement in animal stroke studies (MacLellan et al., 2006). Although much progress has been made in elucidating the pathophysiology of ICH, there remains a lack of successful clinical translation from animal studies to human patients. In clinical trials, therapies are often given at a greatly delayed chronic phase from onset. However, in animal studies, investigations are often conducted in the acute phase after onset, and the oedema, haematoma extension or cell death and other histological alterations in the peri-haematoma are quantified as end points to measure their efficiency (McCracken et al., 2003). As injury to distal regions such as axonal degeneration may contribute to impairment but remain undetected (Corbett and Nurse, 1998), histological changes must be effectively assessed. In addition, because the spontaneous recovery of functional improvement has been observed in a rodent model (MacLellan et al., 2006; Mestriner et al., 2011), the sensitive functional tests of long time course investigations are greatly required. Future studies must include the following items: appropriate animal models that consider the structural difference between animals and human beings, appropriate behavioral tests that accurately demonstrate the progress of disease and recovery in animal models and a good experimental design that considers both the differences in symptoms between human and animals and the important roles of white matter in stroke models.

The classic ICH models include balloon inflation (Mendelow, 1993; Lopez Valdes et al., 2000) and cerebral blood vessel avulsion (Funnell et al., 1990). Two prevalent animal models have been commonly used to mimic human ICH syndrome: the blood infusion (Mendelow et al., 1984; Nath et al., 1986; Rynkowski et al., 2008) and the collagenase-induced models (Rosenberg et al., 1990). Since the first use of an ICH rodent model in the early 1990s, many investigators have tried to identify ideal models that can closely mimic clinical spontaneous ICH and show greater prolonged impairment with lower death rates. Different modified animal models have been developed to consistently detect ICH deterioration in animals, including a high-dose injection of collagenase (MacLellan et al., 2006), an injection of collagenase with heparin administration (Terai et al., 2003), a double injection of blood (Belayev et al., 2003) and an injection of collagenase close to the IC (Masuda et al., 2007a,Masuda et al., 2007b).

The differences between the collagenase and blood injection models are well established. The features of these two classical experimental ICH models are well characterized in terms of their haematoma extension, tissue loss volume, cortex, and white matter atrophy and sensorimotor deficits, based on 0.2 U type IV collagenase and 100 μl autologous tail vein blood injections into the unilateral ST of adult SD rats (MacLellan et al., 2008). Although a greater haematoma size is induced in the blood model during the acute phase 12 h after onset, the tissue loss volume is substantially greater in the collagenase model at 6 weeks after onset. The neuronal losses in both the ipsilateral ST and peri-haematoma region of the ST are greater in the collagenase model. The reductions in corpus callosum volume (white matter atrophy) and cortex thickness (gray matter atrophy) are greater in the collagenase model. In addition, according to the Neurological Deficit Scale (NDS), the collagenase model produces significant sensorimotor deficits that remain impaired at 28 days after onset.

The functional recovery and structural alterations of different doses of collagenase have also been compared (MacLellan et al., 2006). Using 0.06 (mild), 0.12 (moderate), and 0.18 U (severe) of bacterial collagenase type IV infused into the ST of SD rats, MacLellan reported a mortality level of 0 for all groups in an ICH model. The severe group experienced more common extensive injury and greater rostrocaudal involvement of the ST than the other two groups. The long-term (28 days after onset) sensorimotor deficits examined by a battery of behavioral tests have also been observed in severe groups receiving 0.18-U collagenase injections. Furthermore, given the concerns related to the broad range of deficits caused by extensive lesioned structures and the time-dependent progress of symptoms, investigators have recommended different behavioral tests. For instance, the skilled reaching and Cylinder tests are sensitive in detecting lesions in the corpus callosum, IC and ST, and have thus been recommended for evaluating even mild injury. The NDS has been recommended for the early phase after ICH (1 week), and the staircase test has been recommended for detecting long-term deficits.

The current study applied an IC-ICH model to rats. IC models produce long-lasting sensorimotor deficits 28 days after onset, and rarely cause the abnormal functions associated with striatal injury in rats, such as spontaneous circling (Masuda et al., 2007a; Ishida et al., 2011). Furthermore, an increase in the neurogenesis of endogenous neural progenitor cells and newly born neuroblast migration has been observed in IC model rats compared with sham rats (Masuda et al., 2007b). These findings have provided encouraging evidence that the IC model is an appropriate model for translating ICH research. The IC is a common site of ICH in patients, and small lesions in the IC usually lead to severe impairment (Qureshi et al., 2001; Xi et al., 2006; Masuda et al., 2007a). However, data related to the characterisation of IC models is still lacking.

The current study developed an IC lesioned ICH model with a 0.2-U collagenase injection and compared the structural and functional deficits of IC ICH with those of traditional striatal ICH. The presence of a relevant smaller tissue loss volume in the IC group (Figure 1 and Table 1) produced consistent structural changes with long-lasting neurological dysfunction. In the IC model, Figure 1 and Table 1 also showed considerable variation in tissue loss volume measured by MRI and histology over time. The equations used to calculate tissue loss volume showed that in the acute phase of ICH, such volume consists primarily of hematoma expansion and edema. In the delayed phase that follows edema recession and hematoma reabsorption, in contrast, tissue loss volume consists mainly of the lesion volume (such as cavity and cellular debris) and ventricular enlargement resulting from neuronal death and white matter loss. The volume of hematoma in internal capsule measured by MRI decreased gradually during the first 4 weeks after onset (Matsushita et al., 2013). Moreover, Tadashi Masuda’s study showed that in IC lesioned model, a significantly decreased of corticospinal neurons was observed at 14 days after lesion, but not obvious at 7 day after lesion (Masuda et al., 2007a). The gradual degeneration in corticospinal tract after 7 days might help us explain the tissue loss variation in our models. The limitation of MRI in identification of white matter injury and the limitation of histology sections that brain shrinkage occurs during fixation and dehydration, may lead to the our reported discrepancy in tissue loss volume measured on days 14 MRI images and histology sections (Liu et al., 2013). If we could prove the loss of those white matters in IC model rather than ST model from 7 days after onset in our future study, it might further support our hypothesis that white matter damage contributes more in the prolonged functional deficits in IC models.

The IC lesion resulted in more damage to the structures, and the ST, IC and corpus callosum were partially impaired at times. Tissue loss continued over 8 weeks in the two models. However, the tissue loss volume measured from both MRI and histology section of the IC group was smaller than that of the ST group over a 2-month period (Table 1). To determine the long-term functional deficits of the IC group, the functional outcomes of the two ICH groups, including the mNSS, asymmetry forelimb usage and gait parameters, were compared at 56 days after ICH. The impairment of the mNSS and the ratio of contralateral forelimb usage were still significant in the IC group at 56 days compared with the sham group, and the ST group showed no significant difference compared with the sham group. In addition, the asymmetries in the hind paw print area, forepaw pressure, forepaw print area, duty cycle and impaired stand index of the IC group continued to exhibit a more significant difference than in the ST group at 56 days. Consistent depressant SSEPs amplitude of IC group on day 56 also indicated a significant CST injury in IC model.

Protein kinase C is a family of serine/threonine kinases that is known to be involved in a wide variety of pathways critical for neuronal repair, synaptic remodeling, memory, and learning (Nelson et al., 2008; Li et al., 2014). Unlike other PKC isotypes, the conventional gamma isotype of protein kinase C (PKC-γ) is unique as it is expressed exclusively in the brain and spinal cord and its distribution is restricted to neurons in the CNS (Saito and Shirai, 2002). Corticospinal injuries such as brain injury (Omoto et al., 2011), spinal cord injury (nature), and unilateral pyramidotomy (Harel et al., 2013) result in a significant loss of PKCγ level in CST, so it has been used as a marker to assess the structural status of CST in experimental animal models. It has also been shown that PKCγ level is dependent on the degree of motor function in the experimental autoimmune encephalomyelitis (EAE) model and is used as a novel marker to assess the functional status of CST (Lieu et al., 2013).

In this study, the immunohistochemistry of PKCγ clearly demonstrated a nearly complete loss of PKCγ expression in the ipsilateral medullary CST in IC group on day 56 after ICH. Compared to sham group, IC group has a decreased level of PKCγ in ipsilateral CST on days 3 and 56. Compared to ST group, the expression levels of PKCγ in IC group significantly decreased on day 56 in the ipsilateral CST. The Bielschowsky silver staining positive axons were observed in both IC and ST groups, with a decreased density compared with the sham group. Most important, the waveness of axons as well as several axonal retraction bulbs which formed after the fraction of axon fibers with dilated myelin sheaths and enlarged tubular space were still found in IC group rather than ST group on day 56 after ICH, indicating a more severe long-term axonal degeneration in the IC group.

Thus, in the developed IC lesioned ICH model, a relatively smaller lesion volume and tissue loss induced more severe and long-lasting motor dysfunctions, which was consistent with the clinical conditions and findings of a previous animal study (Masuda et al., 2007a).

## Author Contributions

GL and WP contributed in study concept and design. YL, XS, WW, TD, YML, and LA contributed in acquisition of data and statistical analysis. YL, GL, and XS contributed in analysis and interpretation of the data. YL, GL, WW, and TD drafted the paper. WP, GL, and LA critically revised the manuscript for intellectual content.

## Conflict of Interest Statement

The authors declare that the research was conducted in the absence of any commercial or financial relationships that could be construed as a potential conflict of interest.
